# Retraction: The cryoprotectant trehalose could inhibit ERS-induced apoptosis by activating autophagy in cryoprotected rat valves

**DOI:** 10.1371/journal.pone.0201082

**Published:** 2018-07-17

**Authors:** Hongyan Wu, Qing Chang, The PLOS ONE Editors

Following publication of this article [1], the corresponding author informed the *PLOS ONE* Editors that he was not aware of the submission of the manuscript, which used a different email address to his, and he does not stand by the reported content of the article.

The *PLOS ONE* Editors have been unable to obtain the original data or verify that the study was conducted as reported. In light of the concerns raised, the authors and *PLOS ONE* Editors are issuing a retraction of the article.

QC, HW agreed with the retraction.
